# Sorting out the Value of Cruciferous Sprouts as Sources of Bioactive Compounds for Nutrition and Health

**DOI:** 10.3390/nu11020429

**Published:** 2019-02-19

**Authors:** Ángel Abellán, Raúl Domínguez-Perles, Diego A. Moreno, Cristina García-Viguera

**Affiliations:** Phytochemistry and Healthy Foods Lab, Research Group on Quality, Safety and Bioactivity of Plant Foods, Department of Food Science and Technology, CEBAS (CSIC), Campus Universitario de Espinardo 25, 30100 Murcia, Spain; avictorio@cebas.csic.es (Á.A.); rdperles@cebas.csic.es (R.D.-P.); dmoreno@cebas.csic.es (D.A.M.)

**Keywords:** Brassicaceae, elicitation, growing conditions, broccoli, radish, kale pak choi, isothiocyanates

## Abstract

Edible sprouts with germinating seeds of a few days of age are naturally rich in nutrients and other bioactive compounds. Among them, the cruciferous (Brassicaceae) sprouts stand out due to their high contents of glucosinolates (GLSs) and phenolic compounds. In order to obtain sprouts enriched in these phytochemicals, elicitation is being increasing used as a sustainable practice. Besides, the evidence regarding the bioavailability and the biological activity of these compounds after their dietary intake has also attracted growing interest in recent years, supporting the intake of the natural food instead of enriched ingredients or extracts. Also, there is a growing interest regarding their uses, consumption, and applications for health and wellbeing, in different industrial sectors. In this context, the present review aims to compile and update the available knowledge on the fundamental aspects of production, enrichment in composition, and the benefits upon consumption of diverse edible cruciferous sprouts, which are sources of phenolic compounds and glucosinolates, as well as the evidence on their biological actions in diverse pathophysiological situations and the molecular pathways involved.

## 1. Introduction

In the last decades, a growing interest concerning the implications of diet and physical activity on health has occurred in society. This interest lies in the expansion of life expectancy as well as in the improvement in quality of life, and this has led to interventions based on the incorporation of new healthy foods in the human diet. These new foods are envisaged to constitute a valuable source of bioactive healthy nutrients and non-nutrients that would contribute to delaying the onset of a number of chronic and disabling diseases as well as reducing their incidence and severity. In this sense, consumers are demanding a diversified range of foods that provide health benefits and contribute to well-being. For the consecution of this objective, a wide range of plants, crops, and foods have been studied and characterized throughout the recent decades regarding their potential to exert effects on health, according to their nutritional content and bioactive phytochemical composition. Also, many works have paid attention to the bioaccessibility, bioavailability, and bioactivity which will allow, in the near future, validation of their use in the design of new functional ingredients and foods [1].

In this regard, edible sprouts represent a valuable source of diverse micronutrients (vitamins, minerals, and amino acids), macronutrients (proteins, low in carbohydrates, and a high content of dietary fiber), and plant secondary metabolites (mainly phenolic compounds and glucosinolates (GLSs)). Due to this composition, edible sprouts are a valuable vehicle and opportunity to impact health, delivering beneficial bioactive compounds once incorporated in the diet on a regular basis.

From a commercial point of view, a broad spectrum of sprouts and sprouting seeds is available including, but not limited to, soybean, alfalfa, broccoli, radishes, kale, watercress, and peas. This type of fresh product is gaining interest, not only in the field of gourmet and elite cooking or in dedicated nutrition (e.g., vegetarians and health conscious consumers), but also (and consequently) in the food industry, boosted by interest in sprouts as a source of nutrients and healthy secondary metabolites with a really short production time (5–10 days, depending on species or varieties) [2].

Within the current diversity of sprouts and germinates, cruciferous types (which includes sprouts of Brassicaceae, like broccoli, radish, kale, mustards, radishes, or wasabi) are noticed because of their high content of micronutrients, nitrogen–sulfur compounds (glucosinolates (GLSs) and their derivatives, isothiocyanates (ITCs), and indoles) and phenolic compounds (mainly phenolic acids, flavonols, and anthocyanins) [3,4,5].

This review aims to compile and update the available knowledge on the fundamental aspects of production [6], enrichment in composition, and benefits upon consumption of diverse edible raw sprouts as suitable sources of (poly)phenols and GLSs, as well as the currently available evidence on their biological actions in diverse pathophysiological situations and the molecular pathways involved. In this sense, it has been noticed that there is a close linkage between the bioactive composition of cruciferous sprouts and their capacity to act as “phytopharmaceutics” with a valuable contribution to human health.

## 2. Bioactive Secondary Metabolites in Edible Cruciferous Sprouts

As mentioned above, cruciferous sprouts contain non-nutrient/health-promoting compounds, such as diverse types of glucosinolates and phenolic compounds [5]. The biological activity developed by these compounds is mainly due to their antioxidant capacity, which could lower the deleterious consequences of excessively high levels of reactive oxygen species (ROS) in cells and, thus, decrease oxidative stress (OS) by providing cells with molecular tools to combat the imbalance between the production of ROS and the capacity to modulate the redox balance. These properties have direct effects on a number of cellular processes triggered by ROS, which are related to inflammation and oxidative reactions on DNA, proteins, and cell lipids [7]. In addition, to provide further molecular tools to cells to lower OS, many bioactive phytochemicals present in edible sprouts display biological functions that are crucial for the prevention of carcinogenesis processes and other chronic diseases [1] (Table 1).

### 2.1. Phenolic Compounds in Cruciferous Sprouts

Phenolic compounds are a large class of plant secondary metabolites, sourced exclusively from the shikimate-derived phenylpropanoid and/or the polyketide pathway(s), which feature more than one phenolic ring and are devoid of any nitrogen-based functional group in their most basic structural expression. They are mainly represented by flavonoids and phenolic acids of hydroxycinnamic origin in cruciferous sprouts [12] (Table 1). The diversity of structures is related to a variety of properties associated with specific roles in plants, hence their specific distribution.

The physiological relevance of the phenolic compounds in foods and the cruciferous sprout intake may contribute to the positive effects on health of the phenolic metabolites on the different mode of actions that have been widely studied and reported in literature [13].

The formation of a specific bioactive compound depends on diverse variables, like the stress suffered by sprouts during germination, the environmental conditions, or the nutrient supply [12]. In this sense, cruciferous vegetables and their sprouts feature a similar (poly)phenolic profile, composed mainly by phenolic acid derivatives (e.g., sinapic and ferulic acid glycosylated derivatives and caffeoyl-quinic acids) and glycosylated flavonoids (mainly kaempferol and quercetin derivatives, with few or absent isorhamnetins) [14]. When it comes to colored flavonoids, broccoli, radishes, cabbages, and kale sprouts are rich in anthocyanins—most of them highly acylated and glycosylated forms of cyanidin [3,15,16] (Table 1). The interest in anthocyanins has risen in recent years, because of their role in the control of diseases like obesity or diabetes and the possibility of them acting positively on brain function [17].

### 2.2. Glucosinolates in Cruciferous Sprouts

The GLSs, which are essentially unique to Brassicaceae, are secondary metabolites of the stress response, which are located in intact stable forms in plant cells. The general structure of GLSs consists of a glucose molecule linked to a thiohydroximate-*O*-sulfonate and an amino acid [18]. Attending to this cited amino acid group, GLSs are classified into three groups: aliphatic (including a methionine, isoleucine, leucine, or valine derived moiety in their structure), indole (including a tryptophan derived moiety in their structure), and aromatic (including a phenylalanine or tyrosine derived moiety in their structure) [5]. However, GLSs are not biologically active molecules, but the substrate of hydrolysis reactions after the rupture of plant tissues and the contact with the hydrolysis enzyme myrosinase, a plant thioglucosidase normally located into vacuoles in a specialized cell type named myrosin cells. Through this reaction, myrosinase catalyzes the hydrolysis of GLSs to ITCs or indoles, as well as nitriles and epithionitriles [19], depending on the pH, the presence of Fe and epithiospecifier proteins, and other conditions The ITCs are generally produced under physiological pH conditions (pH 6.0–7.0) and are widely known as chemo-preventive and detoxifying agents [20]. The isothiocyanates (ITCs) and thiocyanates present a sulfide group that is united to the carbon with a double (ITCs) or a triple bond (thiocyanates). On the other hand, nitriles lose the sulfide group and in the presence of Fe molecules, can be metabolized to epithionitrile [21].

Brassicaceae sprouts are of special interest as dietary sources of GLSs (Table 1), being glucoraphanin (GR) the most abundant one, and its derived, sulforaphane (SFN), the most abundant ITC in broccoli sprouts. The SFN needs the presence of glutathione (GSH) to be conjugated and to generate the derivative accessible to cells, where it develops its biological functions as a SFN–GSH derivative [22]. Sulforaphane is responsible for the modulation of a number of molecular pathways in cells, which is the basis of its health-promoting attributions. Another GLS present in cruciferous sprouts (e.g., broccoli and kale) at concentrations that allow their interesting biological activity to be seen in vivo is glucoiberin (GIB) (precursor of the ITC iberin, IB), which has beneficial effects on oxidative stress and cancer prevention [2].

Apart from broccoli, red radish sprouts contain high concentrations of glucoraphasatin (4-methyl thio-3-butenyl) and glucoraphenin, which are its major GLSs [23]. Glucoraphenin is hydrolyzed to the ITC sulphoraphene (SFE), a bioactive compound that contributes to the lowering of oxidative stress in cells as well as providing antimutagenic activity against diverse malignant cell types [5].

In addition, cruciferous sprouts have an important presence of indole GLSs. In this sense, indol-3-carbinol may act as a chemopreventive agent which avoids the proliferation of diverse cancer cell lines, acting over a wide range of signaling pathways (hormonal homeostasis, cell cycle progression, and cell proliferation) [24].

Detailed aspects of the health promoting bioactivity of the GLSs, ITCs, and indoles is discussed in more detail in Section 4 (“The Challenges of Including Cruciferous Sprouts in Balanced diets and Personalized Nutrition”).

## 3. Elicitation of Brassicaceae Sprouts to Enhance the Content of Bioactive (Poly)phenols and Glucosinolates

The production of edible sprouts allows the modification of certain pre- and post-harvest conditions to try to improve the production of secondary metabolites, such as GLSs or phenolic acids. Indeed, nowadays, elicitation has been employed in agronomic production to increase the expression of specific genes of interest in plants [25]. The elicitation alternatives that could induce stress in the plants vary from the modification of the abiotic factors affecting sprout growth in the chamber, such as temperature, humidity, and the light intensity/period, to the use of specific biotic elicitors, like plant hormones (methyl-jasmonate and ethylene, among others) or amino acids (methionine) [26]. In this context, elicitors can be classified as biotics (plant hormones, proteins, natural toxins, oligosaccharides, lipopolysaccharides, polysaccharides, or extracts with essential oils) and abiotics (minerals, chemical elements, physical damage, or benzothiadiazole) [6]. Moreover, seed priming before the exogenous elicitation has also been described as modulating the response of the sprouts [6]. Nowadays, these elicitation practices are extensively used to implement the production of edible sprouts, while new emergent agro-technologies, like the use of light-emitting diode (LED) lights to elicit secondary metabolites ((poly)phenols and GLSs) in edible sprouts, has been less explored. In this regard, Baenas et al. (2014) [6] clustered many techniques and their effects on the content of bioactive (poly)phenols and GLSs or the transcription of specific genes in diverse raw edible sprouts, and updated information is presented in Table 2.

The elicitation with Mg (50–300 mg/L) enhanced the production and concentration of total phenolics in radish sprouts when applied at a concentration of 300 mg/L, although regarding broccoli sprouts, it reduced the content of total phenolics when applied at 50 mg/L [27] (Table 2). Besides, the cited study analyzed the influence of different Mg dosages on the defense capacities of broccoli and radish against OS, and significant modifications of the antioxidant capacity were demonstrated with augmented activity of the major antioxidant enzymes (catalase (CAT), gluatathione reductase (GR), and ascorbate peroxidase (APX)). Specifically, the activity of CAT in Mg-enriched sprouts increased in broccoli (up to 46.7% higher), but decreased in radish sprouts (by 1.5–20.0%). On the other hand, the activity of GR increased in radish sprouts (32.0–96.0% higher), while it decreased in broccoli (14.8–40.7% lower). The APX activity increased in broccoli sprouts, but just at intermediate concentrations (50 and 100 mg/L), while in radish sprouts, it significantly decreased (7.6–24.1%). These enzymes are key to the antioxidant capacity of the plants. However, currently, it is not clear how the improvement in the reduction of ROS, as a consequence of the elicitation of Mg, is a positive effect for plants, and further research is required (Table 2).

It is also important to mention that the elicitation with plant hormones can be effective to modify the secondary metabolism of higher plants. In this regard, methyl jasmonate (MeJA) and the free acid associated jasmonic acid (JA) are regulators with key influences on the diverse steps of cellular pathways involved in the development of Brassicaceae sprouts in the stages of seed germination, root growth, fertility, and senescense, among others. However, the succes of the elicitation with MeJA and its influence on the secondary metabolism depends on an array of factors like the presence of induced light [35] or the combination with other elicitors, like polysaccharides [34]. In this sense, Al-Dabhy et al., 2015 demonstrated that light has a decisive influence on the production of GLSs and anthocyanins in radish sprouts at different developmental stages. In fact, when grown under light absence conditions with and without MeJA elicitation, a less intense augmentation of anthocyanins and most GLSs was observed relative to that produced with exposition to light. Besides, the application of MeJA to radish sprouts with induced light showed significant increases mainly represented by glucoraphanin (1.5-fold), glucoerucin (1.6-fold), glucotropaeolin (1.3-fold), 4-hydroxyglucobrassicin (4.4-fold), pelargonidin (1.7-fold), and cyanidin (2.0-fold) (Table 2). Finally, some GLSs (glucoalyssin, glucoerucin, glucotropaeolin, glucoraphasatin, and glucobrassicin) increased their concentration in radish sprouts grown in darkness when the presence of MeJA was not higher than 100 µM [30].

**Figure 1:**
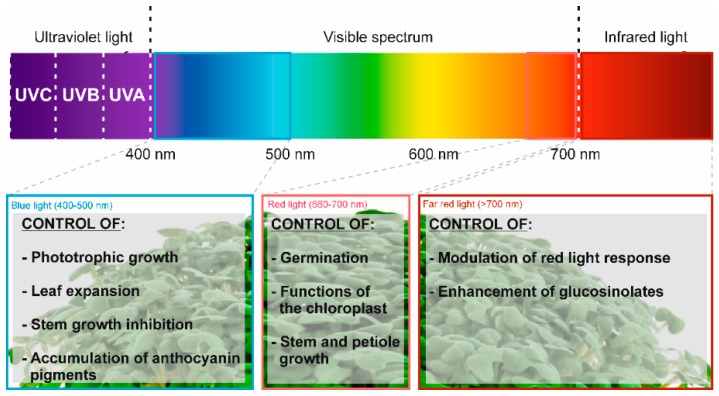
Light spectra influence on the development of kale sprouts.

Light, in addition to being a vital element required for plant survival, constitutes a factor with the capacity to critically influence a range of variations regarding the composition and metabolism observed during sprout growth. In connection to this role, light generates stress in plants and thus, activates specific enzymatic pathways of interest for the production of health-promoting bioactive compounds [25]. In this sense, the wavelength of the spectra applied during the development of seedlings has shown interesting changes. Nowadays, the use of LED lights allows us to apply and characterize the effects on plant growth and composition of all of the spectra, including far-red light (>700 nm). Far-red light has been proven to be a powerful booster that enhances the occurrence of glucosinolates and phenolic compounds in kale sprouts (Figure 1) [32].

Carvalho et al. observed that the application of different light wavelengths (470, 660, and 730 nm) modifies diverse molecular pathways routes in cells, affecting the concentration of bioactive phytochemicals (Table 2), with the most remarkable combination being the use of far-red light with other colors like blue (responsible for the regulation of phenolic compounds) and white, and having the appropriate amount of time in darkness (enhanced the total GLSs by 20.0%), throwing off interesting results compared to the regular application of white light and darkness (Table 2).

## 4. The Challenges of Including Cruciferous Sprouts in Balanced Diets and Personalized Nutrition

Balanced diets are critical for the provision of energy and nutrients essential to human health and well-being. Besides, a balanced nutritional supply should be considered carefully in diverse pathophysiological situations. Under a specific physiological status, a given nutrient supply could constitute a preventive or a risk factor. Anyway, to date, a consensus on the most appropriate dietary patterns has been set up, featuring a high proportion of plant foods to lower the incidence and severity of a number of degenerative pathologies, namely cardiovascular diseases, metabolic disturbances, and tumoral processes. This is of special relevance regarding the specific molecules prone to developing biological functions in humans. Indeed, (poly)phenols and GLSs, in addition to bioactive nutrients, are able to produce diverse effects that go beyond basic nutrition, being active on diverse pathophysiological processes and capable of selectively affecting cell proliferation, apoptosis, inflammation, cell differentiation, angiogenesis, DNA repair, and detoxification [36].

Nowadays, it is well accepted that the consumption of cruciferous sprouts is positive for the prevention of health problems, based on the presence of a number of bioactive secondary metabolites (phytochemicals) that naturally occur in plant foods, which have the capacity to act on diverse molecular targets into cells. This range of molecular mechanisms, which is susceptible to activation or inhibition by the GLSs, ITCs, and (poly)phenols present in cruciferous sprouts triggers diverse pathways governed by the expression of a broad variety of genes. Among them, to date, the following pathways have been identified: the inhibition of the DNA binding of carcinogens, the stimulation of detoxification of potentially damaging compounds, DNA repair, the repression of cell proliferation and angiogenesis (directly related to tumor growth and metastasis), the induction of apoptosis of malignant cells [37,38], and the ability to enhance the antioxidant tools of cells and promote free radical scavenging [39,40]. Regarding this biological activity, the modulation of the inflammatory cascade, and more specifically, the transcription factor NF-κB by GLSs, ITCs, and (poly)phenols, are also involved in the anticancer activity [41]. Hence, hereafter, the evidence on the value of incorporating cruciferous sprouts to regular diets to prevent a number of clinical situations is reviewed (Table 3), and the molecular mechanisms involved are also discussed.

### 4.1. Effect of Cruciferous Sprouts on Type 2 Diabetes Mellitus

Type 2 diabetes mellitus (DM) is characterized by hyperglycemia and abnormal carbohydrate, lipid, and protein metabolism; it is a multifactorial condition triggered by disturbances of insulin activity in peripheral tissues [76]. In mammals, insulin stimulates the disposal of glucose into skeletal muscle and adipose tissue, while lower levels of gluconeogenesis and glyconeogenesis in hepatocytes suppress the release of free fatty acids from adipose tissue and modulate the transport of amino acids to muscle and liver, reducing protein catabolism [40,77]. Plant foods attenuate the severity of DM by enhancing the sensitivity of cells to insulin due to their content in (poly)phenols [78]. In this regard, dietary intervention with broccoli sprouts in DM patients contributes to the reduction of fasting blood glucose and insulin concentration and resistance to almost physiological levels [4].

The effects on DM of cruciferous sprouts were demonstrated by Taniguchi et al. (2006) using Japanese radish sprouts in normal and streptozotin-induced diabetic rats [40]. The ingestion of radish sprouts lowered the plasma levels of fructosamine and glucose with a decrease in the plasma level of insulin, demonstrating that the hypoglycemia caused by the intake of radish sprouts could be not due to an augment of insulin production but is due to an improved sensitivity or an insulin-like activity [40]. Besides, the authors proposed that hypoglycemic activity could be influenced by the radical scavenging activity and thus, the antioxidant potential of the phenolic compounds present in this food. Indeed, the flavonoids improved the insulin sensitivity, allowing a successful hypoglycemic effect [79,80]. More recently, Baenas et al. (2016) studied the metabolic activity of radish sprout-derived ITCs in *Drosofila melanogaster,* demonstrating a decrease in the glucose content in the flies and upregulation of the *spargel* gene (the homolog of the mammalian PPARγ-coactivator 1α), as well as the inhibition of α-amylase and α-glucosidase in vitro [43]. Hence, it was proven that the intake of radish sprouts decreases the glucose content in fruit flies by modulating the energy metabolism.

The metabolic disturbances of DM featured also side complications that could be attenuated by the bioactive phytochemicals present in cruciferous sprouts. In this aspect, it has been noticed the occurrence of vascular complications that are closely related to an event known as “metabolic memory” [81], through which the reduction of sugars leads to in the formation of Schiff bases and Amadory products, and the repeated cycles of dehydration and concentration give rise to advanced glycation end products [82]. The slow ratio of metabolism of these products, as well as their capacity to induce the expression of their own receptors fits the concept of “metabolic memory” [83]. The interaction of advanced glycation end products to their receptor elicits the generation of ROS and triggers the inflammatory cascade, as well as thrombotic and fibrotic reactions in a diverse range of cells and tissues [82]. Such vascular effects associated with DM require the development of new therapeutic approaches that contribute to moderating the severity of these processes [81]. So, it has been revealed that the consumption of cruciferous sprouts provides bioavailable ITCs, including SFN and others. In connection with the bioavailability of ITCs, besides other biological actions, it is inhibited the formation of glycation end products [84], thus decreasing the expression and excretion of biomarkers of inflammation (prostaglandins) and thrombosis (thromboxanes) in humans [45]. These findings suggest a possible contribution of dietary sources of ITCs as preventive agents against the micro- and macro-vascular complications of diabetic conditions [81].

Summarizing the relevance of broccoli sprouts to modulate DM in diabetic processes, to the present date, it has been demonstrated that the consumption of these bioactive-rich foods for a relatively short period (4 weeks), results in a significant decrease in circulating insulin in DM patients [85] by a number of molecular mechanisms, promoting cruciferous sprouts as a valuable food for balanced diets.

### 4.2. Anti-Inflammatory Activity of Cruciferous Sprouts

The available information retrieved from basic and epidemiologic research reveals that, nowadays, it is accepted that secondary metabolites of cruciferous foods prevent inflammation through the capacity to activate the Keap1/Nrf2/ARE pathway [86]. In this aspect, the ITCs are efficient blockers of the cascade of molecular events following the actions of pro-inflammatory stimuli on endothelial cells by modulating the expression of chemoattractant and adhesion molecules or by preventing the phosphorylation and degradation of key kinases involved in the inflammatory pathways, among other mechanisms [87]. These biological functions of ITCs further contribute to the modulation of atherosclerotic events and suppress raised wall thickness, structural derangement, vascular fibrosis, inflammation, oxidative/nitrative stress, and apoptosis [81].

Dietary intervention with broccoli sprouts led to a significant decrease in the plasma levels of C-reactive protein [47], which is produced by hepatocytes in response to a variety of inflammatory cytokines [88]. This was further confirmed by López-Chillón et al. (2018) in an interventional follow-up study aimed at monitoring the anti-inflammatory effect of the daily consumption of broccoli sprouts for 70 days in overweight volunteers with chronic subclinical inflammation and augmented levels of IL-6, TNF-α, and C-reactive protein [46]. In this intervention, the daily ingestion of 30 g of broccoli sprouts resulted in a significant decrease in the plasma levels of IL-6 and C-reactive protein by 38.0% and 59.0%, respectively. The correlation analyses developed by the authors revealed a significant relationship between the decrease of the inflammatory markers and the plasma levels of the bioactive sulfur compounds of broccoli sprouts. The authors speculated that the eventual participation of the Keap1/Nrf2/ARE pathway may also be responsible for the monitored effect [46].

Medina et al. (2014) revealed that the intake of broccoli sprouts constitutes a dietary intervention capable of modulating the level of urinary markers of inflammation and vascular events (prostaglandins and thromboxanes), while OS was not modified in healthy volunteers taking 30 or 60 g of broccoli sprouts [45]. This acute intervention monitored the expression of gold markers by advanced chromatography and spectrometry methods that prompted them to report a decrease in the urinary expression of tetranor-PGEM and 11β-PGF_2α_, as well as 11-dehydro-TXB_2_. These results reinforce previous data on the effects of ITCs as modulators of inflammatory processes, even as a short or acute intervention [45] but would require longer studies. Despite this evidence, to date, controversy remains concerning the anti-inflammatory activity of ITCs because of the gap in knowledge on the NF-κB-dependent transcriptional activity in endothelial cells exposed to these bioactive compounds [81].

### 4.3. Capacity of Bioactive Molecules to Modulate Oxidative Stress (OS)

Cruciferous sprouts have also been cited as valuable sources of natural antioxidants, namely vitamins A, B_6_, C, and K, as well as lutein, zeaxanthin, other carotenoids, and tocopherols [89]. Additionally, an appreciable role of the antioxidant activity of these food matrices is attributed to flavonoids (flavonols and anthocyanins) and phenolic acids as well as sulfur-based compounds (GLSs and ITCs), according to the extensive literature available [89].

Nuclear factor (eryhroid-derived 2)-like 2 (Nrf2) is a transcription factor that is modulated by ITCs and is responsible for the regulation of the redox balance [4]. In addition, inside the cells, the bioactive ITCs (e.g., SFN) interact with Keap1/Nfr2/ARE pathway [86], preventing the risk of cell malignancy and the onset of cancer processes as a consequence of an increased level of ROS [90,91]. In this regard, it has been observed that ITCs decrease lipid peroxidation by up to 18.0% after the consumption of broccoli sprouts [92]. An additional demonstration of the capacity of broccoli sprouts to prevent OS was provided by Zhu et al. (2008), who reported that the oral ingestion of broccoli sprouts, as a dietary source of SFN, protected smooth muscle cells from oxidative injury by inducing the cellular and mitochondrial antioxidants and phase-II enzymes (superoxide dismutase, catalase, reduced glutathione, glutathione peroxidase, glutathione reductase, glutathione-S-transferase, and NAD(PH) quinone oxidoreductase 1) [93].

### 4.4. Enhancing the Consumption of Cruciferous Sprouts to Reduce Carcinogenesis

A range of tumor types affects humans, even though medical treatments have experienced an improvement in the last years. In addition, the newly described anti-cancer therapies are not free of toxic effects that impact negatively on the patients’ health [94]. The current advances in plant secondary metabolites for anti-cancer activities, which have fewer side effects, either by themselves or as coadjutants of anti-tumor therapies, could turn into more efficient therapies. Thus, plant-derived metabolites are good sources of new active anti-cancer drugs with reduced cytotoxicity [95].

The bioactive secondary metabolites in cruciferous sprouts have been characterized as being capable of reducing the incidence of cancer, according to a range of properties, such as anti-proliferation and apoptotic cell death activity [94]. Many times, the induction of cancer processes is based on exposure to xenobiotics, whose removal could contribute to reducing the carcinogenesis risk. In this regard, Melega et al. (2013) studied the capacity of sprout extract from Tuscan black cabbage to metabolize xenobiotic (phase-I and phase-II) enzymes and antioxidant defenses in vivo and demonstrated its capability to modulate the expression and activity pattern of hepatic phase-I cytochrome P450 monooxygenase while increasing the activity of the cell antioxidant machinery [54]. The authors attributed the plethora of molecular effects observed to eventual additive and/or synergistic interactions between the diverse bioactive nutrients and non-nutrients that impact the multiple mechanisms involved in the development and advancement of the multistep carcinogenesis events [54].

The epidemiological evidence available indicates that frequent intake of cruciferous foods is associated with lower incidence of multiple tumor types, due to the capacity of ITCs to interact with the Keap1/Nfr2/ARE pathway [86], contributing to the delay or even the reversal of the development of pre-neoplastic lesions, thereby improving survival rates by acting as ‘therapeutic’ agents to malignant cells [96].

The ITCs phenethyl isothiocyanate (PEITC), benzyl isothiocyanate (BITC), and SFN, have been demonstrated to have chemo-preventive activity in diverse in vitro models, exhibiting multi-target activities in cells and tissues [41]. Early works of Fahey et al. (1997) demonstrated the capacity of broccoli sprouts to reduce the incidence of breast cancer in vivo due to the presence of ITCs acting as inducers of enzymes that protect against carcinogens. It is worth mentioning that broccoli sprouts contain low amounts of indole GLSs (precursor of indol-3-carbinol), which were related to tumor promotion in experiments designed to test extremely high doses that are not representative of a dietary intake [97].

The potential of bioactive compounds of broccoli sprouts to act as anti-proliferative agents against intestinal tumor processes has been attributed, still in a partial extent, to the metabolism of ITCs by the biological machinery of enterocytes and hepatocytes [98,99]. However, as suggested by Baenas et al., (2015), the anti-tumor contribution of ITCs, together with other bioactive compounds present in broccoli sprouts, requires further study, since the higher activity demonstrated with whole extracts, when compared to isolated SFN, may indicate synergic activities [98,99].

Munday et al. (2008), demonstrated the inhibitory effect of broccoli sprouts on urinary bladder carcinogenesis through an in vivo assay that revealed the capacity of freeze-dried aqueous extract of broccoli sprouts to decrease its incidence, multiplicity, size, and progression, induced by *N*-butyl-*N*-(4-hydroxybutyl) nitrosamine in rats [100]. Interestingly, this inhibitory activity was associated (and significantly correlated) with a significant induction of glutathione-*S*-transferase and NAD(P)H-quinone oxydoreductase I in the bladder and was related to the presence of bioactive ITCs in broccoli sprouts that, in bladder cells, in vitro and in vivo, induced the expression and activity of glutathione-*S*-transferase and NAD(P)H:quinone oxidoreductase 1 by up to 3.4 and 2.7-fold, respectively [101]. This evidence further demonstrated the anti-cancer potency of broccoli sprouts, reinforcing previous findings of induced apoptosis and cell cycle arrest in bladder cancer cells, as well as inhibiting angiogenesis, which is associated with tumor progression and metastasis [102,103,104,105,106]. The delivery of ITCs from broccoli sprouts to the bladder during urinary excretion could reduce the incidence of bladder cancer, although the urinary concentration of ITCs capable of developing this protective effect remains to be determined [100].

In another study, Dinkova-Kostova et al. (2010), revealed that feeding mice with broccoli sprouts for 13 weeks with daily doses of 10 μmol of GR protected against the development of skin tumors for 13 weeks and reduced the multiplicity and volume of established tumors by 47.0% and 70.0%, respectively [107]. This finding differs from previous works in terms of the application form of the broccoli sprout extract. In this case, topical application reduced the number of small tumors, but not large tumors, an effect that seemed to be due to the local concentration of the bioactive agent (SFN), as well as the types of derivatives produced as a consequence of the metabolic reaction in diverse cell types and tissues [86]. The authors speculate that feeding mice with broccoli sprouts containing GR protects against skin cancer by unknown mechanisms; however, the available knowledge on the molecular pathways targeted by these compounds suggests that they cause cell cycle arrest and apoptosis and thus, inhibit tumor development. Knatko et al. (2016) demonstrated that the impact of dietary ITCs on the incidence and severity of skin cancer could be related to the protection mediated by the Keap1/Nrf2/ARE pathway applying a mouse model [108]. Recently, a placebo-controlled, randomized clinical trial in which almost 300 volunteers ingested a broccoli sprout-based beverage containing 40 mmol SFN and 600 mmol GR demonstrated that the detoxification of benzene and acrolein mercapturic acids was enhanced by the broccoli sprout beverage by 61.0% and 23.0%, respectively [109]. This result is in agreement with the described capacity of the bioactive compounds in cruciferous sprouts to detoxify environmental carcinogens and toxins.

Once some of the health benefits associated with the consumption of broccoli sprouts had been identified, it was necessary to shed some light on the bioactive compounds responsible for such healthy attributions. In this regard, even though SFN and iberin have been reported as the major ITCs of broccoli sprouts, their functional attributions seem not to be responsible for the biological benefits associated with the consumption of these sprouts. In this aspect, Riedl et al. (2009) performed a placebo-controlled, single blind, dose-escalation trial that included 65 volunteers who ingested from 25 to 200 g of broccoli sprouts homogenates daily (corresponding to 13–102 mmol SFN), for three days [58]. This assay demonstrated a significant correlation between the dietary intake of this ITC and the induction of phase-II mRNA in the lavage fluid of the upper airways. This consequence was attributed to the modulation of the expression of *glutathione-S-transferase Mu-1* (*GSTM1*), *glutathione-S-transferase Pi-1* (*GSTP1*), *NAD(P)H quinone dehydrogenase 1* (*NQO1*), and *HO-1* genes by amounts of broccoli sprouts equal to or higher than 100 g [58] and supports the development of future clinical studies aiming to examine the potential benefits of SFN in modulating allergic respiratory inflammation caused by oxidative insults.

With respect to the anti-cancer mechanisms of action of isothiocyanates, it has been demonstrated that this follows two main routes: a reversible reaction of the electrophile central carbon with cysteine residues in proteins and glutathione towards the formation of thiocarbamate products and irreversible alkylation reactions with amino-groups in N-terminal residues of proteins with the lysine or secondary amines [110]. However, according to the extensive descriptions available in the literature, the main molecular mechanisms involved in the anti-tumor activity of ITCs are represented by their capacity to inhibit the phase-I enzymes cytochrome P-450s, which are, in turn, responsible for the activation of carcinogens and the induction of phase-II detoxifying enzymes, viz. quinone reductase, UDP-glucuronosyltransferase, and glutathione-*S*-transferase, through an Nrf2-dependent pathway [41,86,111,112,113,114,115,116]. The antitumor power of ITCs has also been proven to induce cell cycle arrest and malignant cells apoptosis [117], the generation of ROS [118,119], the capacity to regulate the activation of the STAT3, NF-κB, and Nrf2 transcription factors [111,120,121], and to inhibit Mitogen Activated Protein Kinases (MAPK) and PKC activities [119,122]. However, the capacity of (poly)phenols and GLSs to modulate the activity of Nrf2 should be taken cautiously because this has been recently related to certain side effects, such as cancer cells survival, and resistance to chemotherapeutics and radiotherapy [123].

In addition to the described impact of cruciferous bioactives on malignant cells, most phytochemicals in cruciferous sprouts are able to modify the immunological microenvironment in which tumor cells grow. In this regard, dietary intake of brassica foods may contribute to the regulation of the functions of the immune system, providing additional and innovative options for cancer treatment in the near future [94].

The interest in cruciferous sprouts as dietary sources of bioactive molecules is even higher because of their relatively low production cost, which has boosted a broad multidisciplinary research approach involving ethnopharmacology, botany, pharmacognosy, and phytochemistry in the recent years [124]. However, in humans, there is a strong inter-individual variability that affects the protective actions of the dietary sources of GLSs and ITCs. This diversity is mainly due to variations in the individual intestinal microbiota that might be featured by distinct thioglucosidase activity and the existence of polymorphisms of glutathione transferases, which are responsible for the metabolism of ITCs, among other factors [107].

Epigenetics is regulated by a number of processes, including the methylation of DNA, the modification of histones, and by non-coding microRNA that are prone to regulating cellular proliferation and viability [125]. Closely related to the investigation of the capacity of cruciferous sprouts (and whatever other foods) to prevent the development of cancer are a number of epigenetic studies. In this regard, it has been demonstrated that malignant cells use these epigenetic traits to control growth and metastasis, while, interestingly, bioactive indoles and sulfur-compounds have been revealed as capable of controlling these effects by acting as powerful modulators of DNA methylation and the molecular modification of histones, thus contributing to the prevention of cancer among other pathologies through hormone and non-hormone-based activities. Hence, in general, it has been demonstrated that ITCs and indoles may contribute to the modulation of gene expression related to the metabolism and excretion of xenobiotics, free radical scavenging machinery, regulation of the cellular cycle and apoptosis, and the response of cells against stress [126]. For instance, inducing the demethylation of the *hTERT* control region or by regulating miRNA knockdown, through which augment the apoptosis of malignant [125]. In this regard, SFN has been identified as the most relevant secondary metabolite responsible for such activities, with broccoli sprouts being the main dietary source [127].

## 5. Conclusions and Future Directions

To the present date, a number of studies have encouraged the consumption of cruciferous sprouts as interesting sources of biomolecules with beneficial effects on health, such as anti-cancer, anti-inflammatory, and antioxidant capacities.

In the current literature related to cruciferous sprouts and their health benefits, these foods have been consistently demonstrated as contributors to the normalization of blood glucose levels and the lipid profile, as well as to the maintenance of redox balance in cells and tissues. These functions have a direct effect on the overall health of humans. More specifically, it is doubtless that the bioactive compounds in cruciferous sprouts are beneficial for the treatment of some metabolic disorders, such as DM and its associated vascular complications. In parallel to the control of glycaemia and insulin levels and resistance, these foods have a close connection with the plasma lipid profile that constitutes an additional relevant subject in the promotion of health.

It is also important to highlight that the bioavailable bioactive compounds in sprouts constitute a useful dietary tool for modulating the molecular parameters of specific pathophysiological situations (enhancement of phase II enzymes, modulation of the level of interleukine-6, C-reactive protein, and tumor necrosis factor-α, and inhibition of NF-κB, among others).

On the other hand, the broad information available suggests that the bioactive phytochemicals present in these vegetables have a prominent role in the control of the incidence and severity of a number of cardiovascular processes, contributing to the fine-tuning of dietary habits and to improving human health through the regulation of molecular routes closely related to the onset of a number of diseases. Thus, the development of a number of experimental procedures and epidemiological studies (not only with sprouts but also with cruciferous foods in general) will increase the capacity to prevent and treat health problems with nutrients and phytochemicals.

There is diverse information about the growing conditions for the production of cruciferous sprouts enriched with bioactive compounds (elicitation) to potentiate the biological functions described.

As a general conclusion, it should be mentioned that caution is required when reporting the biological benefits of cruciferous food intake because of the lack of consistent demonstration of the benefits of incorporating these foods into the dietary habits due to the high inter-individual variability of the parameters monitored as well as to the heterogeneity of the interventions (sampling schedules, doses, sample size, etc.) that do not allow us to outline the effective dosages or concentrations of bioactives that should be recommended to achieve the desirable benefits with cruciferous sprouts to take real advantage of the bioactive (poly)phenols and GLSs present in cruciferous foods.

## Figures and Tables

**Table 1:** The main bioactive phytochemicals and health promoting activities of diverse raw edible sprouts.

Edible Sprout	Main Bioactive Compounds	Main Bioactivities Associated with Sprout Consumption	References
Broccoli (*Brassica oleracea* var. *Italica*)	**Flavonoids** Quercetin, kaempferol, and flavonol glycosides	Cancer risk (↓) Degenerative diseases (↓) Obesity-related metabolic disorders (↓) Allergic nasal symptoms (↓) Inflammation (↓) Pain (↓) Antioxidant capacity (↑)	[5,8]
	**Phenolic acids** Chlorogenic, sinapic, and ferulic acid derivatives		
	**Glucosinolates** Glucoraphanin, glucoiberin, glucoraphenin, glucobrassicin, 4-hydroxyglucobrassicin, 4-methoxyglucobrassicin, and neoglucobrassicin		
	**Isothiocyanates** Sulphoraphane, iberin, and indole-3-carbinol		
Radish (*Raphanus sativus L.*)	**Flavonoids** Quercetin	Risk of cancer (↓) Heart disease (↓) Diabetes (↓) Antioxidant capacity (↑)	[9]
	**Phenolic acids** Ferulic, caffeic and *p*-coumaric acids, and derivatives		
	**Glucosinolates** Glucoraphenin, dehydroerucin, glucobrassicin, and 4-methoxyglucobrassicin		
	**Isothiocyanates** Sulforaphene, sulforaphane, and indole-3-carbinol		
Kale (*Brassica oleracea* var. *acephala*)	**Flavonoids** Quercetin and cyanidin	Risk of cancer (↓) Heart disease (↓) Diabetes (↓) Antioxidant capacity (↑)	[10]
	**Phenolic acids** Chlorogenic and ferulic acids		
	**Glucosinolates** Glucoraphanin, glucoiberin, gluconapin, gluconasturtin, progoitrin, gluconapin, gluconapoleiferin, sinigrin, glucobrassicin, 4-hydroxyglucobrassicin, 4-methoxyglucobrassicin, and neoglucobrassicin		
Pak choi (*Brassica rapa* var. *chinensis*)	**Flavonoids** Kaempferol, quercetin, and isorhamnetin glucosides	Risk of cancer (↓) Heart disease (↓) Diabetes (↓) Antioxidant capacity (↑)	[10,11]
	**Phenolic acids** Ferulic, sinapic, caffeic, and *p*-coumaric acids, and derivatives		
	**Glucosinolates** Gluconapin, glucoalyssin, gluconasturtin, progoitrin, glucobrassicin, 4-hydroxyglucobrassicin, 4-methoxyglucobrassicin, and neoglucobrassicin		

**Table 2:** Compounds of interest in edible sprouts through different elicitors (update from original table of Baenas et al., 2014 [6]).

Raw Edible Sprout	Elicitor Treatment	Elicitor Classification	Application	Target Compound and Increase	Reference
Broccoli sprouts (*Brassica oleracea*) (7 days of growth)	Sucrose, fructose, and glucose (146 mM)	Biotic elicitor	In 0.5% agar media for 5 days after sowing seeds	Total anthocyanins (10.0%)	[28]
Broccoli sprouts (*Brassica oleracea*) (7 days of growth)	Sucrose and mannitol (176 mM)	Biotic elicitor	Hydroponic system for 5 days after sowing seeds	Total anthocyanins (40.0%) and phenolics (60.0%) Total glucosinolates (50.0%)	[28]
Broccoli (*Brassica oleracea*) (7 days of growth)	Met (5 mM) Trp (10 mM) SA (100 μM) MeJA (25 μM)	Biotic elicitors (Met, Trp, and plant hormones—SA and MeJA)	Daily exogenous spraying during 3, 5, and 7 days	Met: glucoiberin, glucoraphanin, and glucoerucin (30.0%) Trp: 4-hydroxyglucobrassicin, glucobrassicin, 4-Methoxyglucobrassicin, and neoglucobrassicin (80.0%) SA: 4-hydroxyglucobrassicin, glucobrassicin, 4-Methoxyglucobrassicin, and neoglucobrassicin (30.0%) MeJA: 4-hydroxyglucobrassicin, glucobrassicin, 4-Methoxyglucobrassicin, neoglucobrassicin (50.0%)	[29]
Broccoli sprouts (*Brassica oleracea*)	Sucrose (146 mM)	Biotic elicitor	In 0.5% agar media for 5 days after sowing	Total GLS (2.0-fold)	[28]
Broccoli sprouts (*Brassica oleracea*) (7 days of growth)	Mg (300 mg L^−1^)	Abiotic elicitor	Suplementation with MgSO_4_	Increase of total ascorbic acid contain (29.1–44.5%)	[27]
Radish sprouts (*raphanistrum subsp. sativus*) (12 days of growth)	MeJA (100 μM)	Biotic elicitor (plant hormones—MeJA)	Treatment with MeJA in growth chamber under dark conditions	Glucoalyssin (1.4-fold) Glucoerucin (2.0-fold) Glucotropaeolin (1.8-fold) Glucoraphasatin (1.4-fold)	[30]
Radish sprouts (*raphanistrum subsp. sativus*) (12 days of growth)	MeJA (100 μM) Light	Biotic elicitor (plant hormones—MeJA-) Abiotic elicitor	Treatment with MeJA in growth chamber under light	Glucoraphanin (1.5-fold) Glucoerucin (1.6-fold) Glucotropaeolin (1.3-fold) 4-hydroxyglucobrassicin (4.4-fold) Pergonidin (1.7-fold) Cyanidin (2.0-fold)	[30]
Radish sprouts (*raphanistrum subsp. sativus*) (7 days of growth)	Mg (300 mg L^−1^)	Abiotic elicitor	Supplementation with MgSO_4_	Phenolic compounds (13.9–21.7%)	[27]
Radish sprouts (*raphanistrum subsp. sativus)*	NaCl (100 mM)	Abiotic elicitor	In 0.5% agar media for 3.5 and 7.0 days after sowing	Total phenolics (30 and 50% in 5 and 7 day-old sprouts, respectively) Total GLS (50% and 120% in 5 and 7 day-old sprouts, respectively)	[31]
Pak Choi sprouts (*rapa subsp. chinensis*)	Application of different wavelengths of LED light (white, blue, and red)	Abiotic elicitor	Medium of perlite for 5 days in darkness and 18 h at the different wavelengths	Total carotenoid content (12.1% and 9.2% with white light (respect to blue and red light, respectively)	[25]
Pak Choi sprouts (*rapa subsp. chinensis*)	Application of different wavelengths of LED light (white, blue, and red)	Abiotic elicitor	Medium of perlite for 5 days in darkness and 18 h at the different wavelengths	Enhanced transcription of genes involved in carotenoid biosynthesis (*CYP97A3, CYP97C1, βLCY, εLCY, β-OHASE1, PDS, PSY, VDE, ZEP*)	[25]
Kale Sprouts (*oleracea var. sabellica*)	Application of different light wavelengths (470, 660, and 730 nm)	Abiotic elicitor	Seeds stratified for 2 days, exposed to light for 1 h, exposed to darkness for between 1 and 3 days and later, the specific light treatment	Total GLS content (31.7%)	[32]
Radish, Chinese kale and pak choi sprouts (3 days of growth)	Glucose (5 g 100 mL^−1^)	Biotic elicitor	Hydroponic system for 3 days after sowing seeds	Total phenolics (20.0%), gluconapin (150.0% and 60.0% in Chinese kale and pak choi, respectively), glucobrassicanapin (110-fold in pak choi)	[33]
Different Brassica sprouts (broccoli, turnip, and rutabaga)	MeJA (25 μM) JA (150 μM) Sucrose (146 mM)	Biotic elicitors (Sucrose and plant hormones—MeJA and JA)	Sprayed for 5 days before harvest	Total GLS (>50%, broccoli; >20.0% turnip; >100.0% rutabaga)	[34]
Radish sprouts (*raphanistrum subsp. sativus*) (8 days of growth)	MeJA (25 μM) SA (100 μM) Glucose (277 mM)	Biotic elicitors (glucose and plant hormones—MeJA and JA)	Sprayed for 5 days before harvest	Total GLS (20.0%)	[34]

Genes: *CYP97A3*: *cytochrome P450 97A3*; *CYP97C1*: *cytochrome P450 97C1*; *βLCY*: *β-cyclase*; *εLCY*: *ε-cyclase*; *β-OHASE1*: *β-carotene hydroxylase 1*; *PDS*: *phytoene desaturase*; *PSY*: *phytoene synthase*; *VDE*: *violaxanthin de-epoxidase*; *ZEP*: *zeaxanthin epoxidase*. GLS: glucosinolates; JA: jasmonate or jasmonic acid; LED: diode electric light; MeJA: methyl jasmonate; Met, methionine; Mg, magnesium; SA, salicylic acid; Trp, tryptophan.

**Table 3:** Demonstrated health benefits of cruciferous sprouts under a range of pathophysiological conditions.

Matrix	Pathophysiological Condition	Effect	Model	Action Mechanism ^Z^	Ref.
Broccoli sprouts	Metabolic profile	No specific effect monitored	Humans	FA 14:1, FA 16:1, FA 18:1, FA 14:0, FA 16:0, FA 18:0, dehydroepiandrosterone, glutathione, cysteine, and glutamine (↑) Deoxy-uridin monophosohate (↓)	[42]
Radish sprouts	Energy metabolism	Decrease glucose level	*Drosophila melanogaster*	Expression of *spargel* (↑)	[43]
Broccoli sprouts	Pregnancy	Prevention of brain injury in newborns	Rats	Not determined	[44]
Broccoli sprouts	Inflammation and oxidative stress	Modulation of inflammation and vascular events	Humans	Not determined	[45]
Broccoli sprouts	Inflammation in overweight population	Anti-inflammatory activity	Humans	IL-6 and C-reactive protein (↓)	[46]
Broccoli sprout powder	Diabetes	Anti-inflammatory effect	Humans	C-reactive protein (↓)	[47]
Broccoli sprouts	Hypertension	Does not improve endothelial function of hypertension in humans	Humans	Not determined	[48]
Broccoli sprouts	Hypertension	Attenuation of oxidative stress, hypertension, and inflammation	Rats	Not determined	[49]
Rutabaga sprouts	Thyroid function and iodine deficiency. Role as goitrogenic foods	Protective effect against thyroid damage Goitrogenic activity not discarded	Male rats	Dietary source of iodine GPX1, GPX3, and FRAP (↓)	[50]
Broccoli sprouts	Hepatic and renal toxicity	Antioxidant activity	Female rats	Phase-II enzymes (↑) Lipid peroxidation and apoptosis (↓)	[51]
Broccoli sprouts	Bowel habits	Decrease in the constipation scoring system Decrease of *Bifidobacterium*	Humans	Not determined	[52]
Broccoli sprouts	Pain assessment and analgesia	Dose-dependent nociceptive activity	Rats	Agonists of central and peripheral opioid receptors	[53]
Tuscan black cabbage sprout extract	Xenobiotic metabolism and antioxidant defense	Improvement of the detoxification of xenebiotics	Rats	Induction of phase-II enzymes and boosting of the enzymatic activity of catalase, NAD(P)H:quinone reductase, glutathione reductase, and glutathione peroxidase	[54]
Japanese Radish Sprout	Diabetes	Decrease in plasma fructosamine, glucose, and insulin in diabetic rats	Rats	Not determined	[40]
Radish sprouts	Diabetes	Increase in blood glucose, triglycerides, total cholesterol, low-density lipoproteins, and very low density lipoproteins	Rats	Not determined	[55]
Broccoli sprout extracts	Skin disorders	Induction of phase-II response	Mice and humans	NQO1 enzyme activity (↑)	[56]
Broccoli sprout extracts	Skin disorders	Protection against inflammation, edema, and carcinogens in humans	Humans	Phase-II enzymes (↑) NQO1 enzyme activity (↑)	[57]
Broccoli sprout homogenate	Physiological upper airway	No specific effect monitored	Humans	Phase-II enzymes (↑)	[58]
Broccoli sprouts	Physiological upper airway	No specific effect monitored	Humans	Nrf2 activity (↑) Secretory leukocyte protease inhibitor (↑)	[59]
Broccoli sprout extract	Asthma	Blocking the bronchoconstrictor hyperresponsiveness of some asthmatic phenotypes	Humans	Activity of Nrf2 regulated antioxidant and anti-inflammatory genes (↓)	[60]
Broccoli sprout extract	Hepatic disturbances	Improvement of liver functions and reduction of oxidative stress	Rats	Not determined	[61]
Broccoli sprout-based supplements	General carcinogenic processes	Chemopreventive effect	Humans	Not determined	[62]
Broccoli sprout extract	Head and neck squamous cell carcinoma	Chemopreventive activity of sulforaphane against carcinogen-induced oral cancer	Mice	Time and dose dependent induction of Nrf2 and Nrf2 target genes (*NQO1* and *GCLC*) Dephosphorilation of pSTAT3	[63]
Broccoli sprouts homogenate	Sickle cell disease (hemoglobinopathy)	Change in the gene expression levels	Humans	Expression of Nrf2 targets (*HMOX1* and *HBG1*) (↑)	[64]
Broccoli sprouts	Oxidative stress	Improvement in cholesterol metabolism and decrease in oxidative stress	Humans	Not determined	[65]
Broccoli sprouts	General carcinogenic processes	Chemopreventive agent	Humans	Histone deacetylase activity (↓)	[66]
Broccoli sprouts	Unspecific frame	Not determined	Humans	Histone deacetylase activity (↓)	[67]
Broccoli sprouts	Antimicrobial activity against *Helicobacter pylori*	Reduction of *Helicobacter pylori* colonization in mice Enhancement of sequelae of *Helicobacter pylori* infection in mice and humans	Mice and humans	Not determined	[68]
Broccoli sprout extract	Allergic response	Broccoli sprouts reduce the impact of particulate pollution of allergic disease and asthma	Humans	Not determined	[69]
Broccoli sprout extract	Prostate cancer	Inconclusive	Humans	Not determined	[70]
Broccoli sprout and myrosinase-treated broccoli sprout extracts	Chemoprevention of carcinogenesis processes	Inconclusive	Humans	No dose response was observed for molecular targets	[71]
Broccoli sprout extract	Psychiatric disorders	Improvement of the cognitive function in patients affected by schizophrenia	Humans	Not determined	[72]
Broccoli sprout extract	Type II diabetes	Reduction of fasting blood glucose and glycated hemoglobin	Mice	(↑) Nuclear translocation of Nrf2 (↓) Glucose production and intolerance	[73]
Broccoli sprout extract	Neurological disorder	Inconclusive improvement of Autism symptoms	Humans	(↑) Gene transcription in multiple cell signaling pathways	[74]
Broccoli sprout homogenate	Viral infections	Enhancement of antiviral defense response	Humans	Modulation of natural killer cell activation Production of granzyme B by natural killer cells (↑)	[75]

^Z^ FA, fatty acids; FRAP, ferric reducing activity of plasma; GCLC, glutamate-cysteine ligase catalytic subunit; GPX1, cytosolic glutathione peroxidase-1; GPX3, cytosolic glutathione peroxidase-3; HBG1, Hemoglobin subunit gamma 1; HMOX1, heme oxygenase (decycling) 1; IL-6, interleukina 6; NAD(P)H, nicotinamide adenine dinucleotide phosphate; NQO1, NAD(P)H:quinone oxidoreductase 1; TNF-α, tumor necrosis factor-alpha; Nrf2, nuclear factor erythroid 2–related factor 2; pSTAT3, signal transducer and activator of transcription-3; TSH, thyroid stimulating hormone. (↓↑) Non-significant variation, (↓) decrease, and (↑) increase.
